# Muscle, Fat, Bone, and Lungs: Unlocking the Fitness and Health Equation of Firefighters in Porto, Portugal

**DOI:** 10.3390/life15030334

**Published:** 2025-02-21

**Authors:** Giorjines Boppre, João Pedro Rocha Nunes, David Gomes Fernandes, Bruno João de Castro Sousa e Ribeiro Carlos, João Miguel Neves Barros, Aline Teixeira Maia de Freitas, José Augusto Rodrigues dos Santos, Rodrigo Zacca

**Affiliations:** 1 Research Center in Physical Activity, Health and Leisure (CIAFEL), Faculty of Sports, University of Porto (FADEUP), 4200-450 Porto, Portugal; giorjines.boppre@gmail.com (G.B.); joaonunes256.jn@gmail.com (J.P.R.N.); davidgf23@gmail.com (D.G.F.); brunojoao462003@gmail.com (B.J.d.C.S.e.R.C.); jmnbarros5@gmail.com (J.M.N.B.); alinefreitasmaia@gmail.com (A.T.M.d.F.); 2 Laboratory for Integrative and Translational Research in Population Health (ITR), 4050-600 Porto, Portugal; 3 Nucleus of Research in Human Movement Science, Universidad Adventista de Chile, Chillán 3780000, Chile; 4 Center of Research, Education, Innovation and Intervention in Sport (CIFI2D), Faculty of Sport, University of Porto, 4200-450 Porto, Portugal

**Keywords:** occupational health, body composition, bone mineral density, pulmonary function, handgrip strength

## Abstract

Background: Firefighters face significant physical demands, necessitating optimal fitness and health monitoring. This study examined the relationships between body composition, bone mineral density, handgrip strength, and pulmonary function in professional firefighters in Porto, Portugal. Methods: Sixty-eight firefighters underwent assessments, including anthropometry, dual-energy X-ray absorptiometry (DEXA) for body composition and bone density, handgrip dynamometry, and spirometry for lung function. Results: 36.5% of participants were overweight, and 33.3% had obesity (Grade 1). Men exhibited greater muscle mass, bone density, and handgrip strength (48.7 ± 7.8 kg vs. 31.6 ± 3.6 kg) compared to women. Spirometry revealed normal lung function in 95.2% of participants, though 20.6% demonstrated handgrip strength values below the risk threshold, indicating vulnerability to reduced physical fitness and an increased risk of injury. Significant correlations were observed between lean mass and both handgrip strength (ρ = 0.551, *p* < 0.001) and pulmonary function, including forced vital capacity (ρ = 0.465, *p* < 0.001). Conclusions: This study underscores the role of body composition, muscle strength, and pulmonary function in firefighters’ health and safety. These findings suggest that these factors are linked to physical fitness and may influence overall health outcomes. Interventions focusing on improving strength and managing body weight could help to reduce health risks and enhance firefighter well-being.

## 1. Introduction

The firefighters of Portugal play a fundamental role in the country’s civil protection, being responsible for a wide range of emergencies, including forest and urban fires, road accidents, floods, and general rescues. In 2024, the number of firefighters in Portugal was significant, with around 35,480 firefighters mobilized in various incidents, representing 80.4% of all civil protection agents involved in emergency situations [1]. These professionals can be classified as volunteer firefighters, professional firefighters, or mixed, and their duties require high physical and psychological preparation. To be considered a professional firefighter, it is necessary to undergo a rigorous selection process and initial training that includes physical and psychological tests and a year of practical experience [2].

Firefighting is one of the main functions of firefighters, especially during the summer when wildfires become more frequent. Furthermore, they are involved in rescues from road accidents, pre-hospital care, mass evacuations, and support during natural disasters [3]. However, firefighters face significant challenges, such as the risk of physical and mental exhaustion, exposure to risks like smoking and stress, as well as other factors that compromise their health [4,5]. Smoking, for example, is more prevalent among firefighters than in the general population, being a risk factor for respiratory and cardiac diseases [6].

Physical fitness is crucial for the effective physical fitness of firefighters’ duties, especially in fighting forest fires and transporting heavy equipment over rugged terrain [7]. Good physical conditioning, including cardiopulmonary fitness and muscular strength, is essential not only to ensure the effectiveness of operations but also to protect the health of professionals, preventing injuries and illnesses [8]. Body composition also plays an important role in physical fitness, with studies indicating that physical fitness can reduce the risk of injuries and improve the quality of life for these professionals [9,10].

Although physical fitness is widely studied in the context of firefighters, there is a significant gap in research on the relationship between body composition, muscle strength, lung function, and bone health of firefighters [11]. Bone mineral density, an important indicator of bone health, can be influenced by factors such as mechanical stress generated by physical activity, body composition, and metabolic disorders [12]. The lack of specific studies on these aspects highlights the need for research that explores how these factors impact work effectiveness, firefighter safety, and disease prevention. Given this, this study aims to examine the relationships between body composition, bone mineral density, muscle strength, and pulmonary function in firefighters from the Porto district.

## 2. Materials and Methods

### 2.1. Sample Recruitment

The sample consisted of 68 professional firefighters belonging to the National Authority for Emergency and Civil Protection (ANEPC), operating in the District of Porto, Portugal. These individuals were selected based on rigorous criteria that ensured the representativeness and integrity of the collected data. The inclusion criteria for participation in the study required that the firefighters be working in the Porto district, perform their duties as professional firefighters, and possess the necessary fitness to carry out all the physical and functional assessments outlined in the study protocol. Moreover, it was essential that all participants were professional firefighters, ensuring they had the appropriate experience and training for the activities carried out during the study. On the other hand, exclusion criteria were established to ensure the safety of the participants and the validity of the results. Firefighters who presented any acute injury, chronic condition, or disorders that could compromise their ability to fully participate in the assessments, such as musculoskeletal disorders or cardiovascular diseases, were excluded from the sample. The participants were informed about the objectives of the study, the assessment methods that would be used, and the possible risks involved, receiving this information in a clear and understandable manner, agreeing to participate in the study, and their understanding of the terms and conditions involved.

### 2.2. Local Assessments and Variables of Interest

The assessments and data collection were conducted at the laboratory of the Research Center for Physical Activity, Health, and Leisure (CIAFEL), located within the premises of the Faculty of Sports of the University of Porto (Porto, Portugal). This study focused on four main variables of interest: (i) anthropometry; (ii) body composition; (iii) muscle strength; and (iv) pulmonary function.

#### 2.2.1. Anthropometric and Sample Characteristics

Body weight was measured with a digital scale (model 899, Seca, Hamburg, Germany), while height was assessed using a portable stadiometer (model 213, Seca, Hamburg, Germany). Based on these measurements, the body mass index was calculated by dividing body weight by height squared (kg/m^2^) [13]. Smoking status (smokers, non-smokers, and ex-smokers [i.e., ≥6 months]) was recorded through anamnesis, where participants self-reported whether they were current smokers, had previously smoked, or had never smoked. A health history questionnaire was also administered to gather information on participants’ medical backgrounds, including the presence of pulmonary or other chronic diseases. Prior to measurement, participants were instructed to empty their bladder and refrain from eating a large meal or engaging in vigorous exercise at least two hours before the measurement to ensure consistency.

#### 2.2.2. Body Composition

Body composition was assessed using the dual-energy X-ray absorptiometry (DEXA) technique with the Hologic Explorer QDR model (Hologic, Inc., Marlborough, MA, USA), under the supervision of a highly skilled technician. All participants were instructed to wear light clothing made of thin material and to remove any external objects, such as jewelry and other metals, to minimize potential interference in the measurements. The positioning of the participants was carried out according to the manufacturer’s standard recommendations [14]. Additionally, participants were instructed to avoid eating a large meal or engaging in strenuous physical activity for at least 4 h prior to the DEXA scan to minimize variations in body composition measurements. The whole-body analysis allowed for the assessment of total fat mass and lean mass, while bone mineral density was specifically measured in the region of interest, the femoral neck.

#### 2.2.3. Muscle Strength

The handgrip strength was assessed using a manual hydraulic dynamometer, model Jamar Plus-Digital and Hydraulic Hand Dynamometer (Warrenville, IL, USA). The test was conducted in accordance with the technical standards [15]. The participants were instructed to remain seated in a chair (without arms) with an upright spine, maintaining a knee flexion angle of 90°, the shoulder positioned in adduction and neutral rotation, the elbow flexed at 90°, and the forearm in half-pronation with a neutral wrist, allowing for up to 30° degrees of extension. The protocol used was one maximum strength repetition for each side. Initially, a familiarization session with the dynamometer was conducted to determine the most comfortable position for the test and, when necessary, adjust the grip size, followed by the measurements. For the execution of the test, they were instructed to press with maximum force for 5 s. The highest value (HGPeak) obtained was computed for analysis. For the assessment of overall muscle strength/weakness (risk threshold), according to age, height, and gender, we use the normative data proposed for professionals in the Portuguese automotive industry [16]. These professionals are required to use their upper limbs a lot, which to some extent is similar to what is required of firefighters.

#### 2.2.4. Pulmonary Function

For the analysis of lung function, the Forced Spirometry Protocol was used to access the forced expiratory volume in the first second (FEV1), the forced vital capacity (FVC), the FEV1/FVC ratio, known as Tiffeneau index (TI), and the Peak Expiratory Flow (PEF). The equipment used was the Spirodoc (Schiller, Rome, Italy). Some important criteria were used to enable individuals’ participation in the protocol, such as: not smoking 24 h before the exam, not consuming large meals before the exam, not doing rigorous exercises before the exam, and not wearing uncomfortable clothes for the exam. The examination protocol consisted of taking a deep breath followed by a maximum expiration and a maximum inspiration, and using a nasal clip and disposable turbine. The forced expiratory maneuvers were performed after a brief demonstration and training, aiming to meet the criteria for acceptability of the curves and reproducibility, requiring three acceptable ones. A minimum of three attempts and a maximum of eight attempts for the test were stipulated, with the best three being selected, and among them, two were computed for repeatability analysis. The criteria used for eligibility included a forced expiration maneuver for six seconds or until the expiratory flow reached a plateau of at least one second. Additionally, at least two attempts with reproducible curves, differing by less than 150 mL between them, were also considered valid, following the criteria established by the American Thoracic Society (ATS) [17]. The predicted values used belong to the database of The Global Lung Function Initiative [18].

### 2.3. Statistical Analysis

The sample size was intentionally and conveniently selected, focusing on professional firefighters (ANEPC, Distrito do Porto, Portugal). Descriptive analyses and partial correlations were performed to investigate the relationships between anthropometric, body composition, and pulmonary function variables, controlling for age, gender, and BMI. Descriptive statistics were first computed to summarize the characteristics of the variables of interest, and normality was assessed using the Shapiro–Wilk test. Partial correlations were then performed to explore the associations between body mass (kg), handgrip strength (right, left, and best value), total bone mass (kg), fat mass (kg), lean mass (kg), fat-free mass (kg), body fat percentage, lung function parameters (FVC, FEV1, PEF), and femoral neck measures (e.g., bone mass, mineral density, T-scores, and Z-scores). A second set of partial correlations examined the relationships between lung function variables (FVC, FEV1, IT, PEF) and trunk mass and composition (total trunk mass, fat mass, lean mass, and lean bone mass). All partial correlations were controlled for age, gender, BMI, and smoking status. Correlation coefficients were interpreted as follows: <0.40 (weak), 0.40–0.75 (reasonable to good), and >0.75 (excellent) [19]. Only correlations with values ≥0.40 were reported and thus discussed. Statistical analyses were performed using R (version 4.4.0, R Foundation for Statistical Computing, Vienna, Austria) with the “psych,” “car,” and “correlation” packages.

## 3. Results

Table 1 presents the data on anthropometric, body composition, bone health, muscle strength, and pulmonary function variables. The average age was 31.4 years for females and 34.2 years for males. Females had an average height of 163.0 cm, and males averaged 173.9 cm. Body weight averaged 66.1 kg for females and 83.7 kg for males. The mean BMI was 25.0 kg/m^2^ in females and 27.7 kg/m^2^ in males. In terms of BMI classification, four females (6.3%) and 15 males (23.8%) were classified as normal weight, three females (4.8%) and 20 males (31.7%) as overweight, and one female (1.6%) and 20 males (31.7%) as obese. Total lean mass was 42.5 kg in females and 61.5 kg in males. Total bone mineral content was, on average, 2.2 kg in females and 2.6 kg in males. Total fat-free mass was 44.7 kg in females and 64.1 kg in males. Total fat mass was 21.0 kg in females and 19.0 kg in males. Body fat % was 31.7% in females and 22.1% in males.

For bone health, the femoral neck area was 4.7 cm^2^ in females and 5.5 cm^2^ in males. Femoral neck bone mass was 4.0 g in females and 5.0 g in males. Femoral neck BMD was 0.8 g/cm^2^ in females and 0.9 g/cm^2^ in males. The femoral neck T-score was 0.1 in females and −0.3 in males. Femoral neck Z-scores were 0.1 in females and 0.1 in males. In muscle strength, right-hand handgrip strength was 31.2 kg in females and 47.3 kg in males, while left-hand HG was 30.4 kg in females and 47.0 kg in males. HGPeak was 31.6 kg in females and 48.7 kg in males.

For pulmonary function, FVC was 4.1 L in females and 5.3 L in males. FVC percentage was 116.1% in females and 111.4% in males. FEV1 was 3.4 L in females and 4.3 L in males. FEV1 percentage was 110.6% in females and 106.9% in males. The TI was 83.0% in females and 80.6% in males. PEF was 6.1 L/s in females and 9.2 L/s in males, with PEF percentage at 88.8% in females and 98.1% in males. Regarding smoking status, the results showed that 4.8% of females and 42.9% of males were smokers, 7.9% of females and 31.7% of males were non-smokers, and 12.7% of males were ex-smokers. No females were reported as ex-smokers. As for health status, only three participants reported a history of mild flu-like symptoms, and one participant had a history of childhood asthma, which has been well-controlled for many years without the need for medication.

The partial correlations (controlled for age, gender, BMI, and smoking status) (Table 2) revealed a moderate to strong positive correlation between handgrip strength and total lean mass (ρ = 0.535, *p* < 0.001), as well as fat-free mass (ρ = 0.547, *p* < 0.001). An inverse moderate correlation was found between handgrip strength and body fat percentage (ρ = −0.325, *p* = 0.012). Additionally, handgrip strength showed a strong positive correlation with pulmonary function parameters, including FEV1 (ρ = 0.529, *p* < 0.001) and PEF (ρ = 0.457, *p* < 0.001). FVC was positively correlated with both total lean mass (ρ = 0.483, *p* < 0.001) and fat-free mass (ρ = 0.471, *p* < 0.001). Similarly, FEV1 was positively correlated with both total lean mass (ρ = 0.476, *p* < 0.001) and fat-free mass (ρ = 0.506, *p* < 0.001), further supporting the relationship between body composition, muscle strength, and pulmonary function.

Table 3 shows the results of the correlations between the variables assessed in the trunk region. Total trunk mass was positively correlated with FVC (ρ = 0.610, *p* < 0.001; “moderate to good”) and FEV1 (ρ = 0.540, *p* < 0.001; “moderate to good”). The same positive correlation was found for lean trunk mass, which correlated with FVC (ρ = 0.555, *p* < 0.001; “moderate to good”) and FEV1 (ρ = 0.574, *p* < 0.001; “moderate to good”), indicating that individuals with greater lean mass exhibited better respiratory function. Trunk fat mass was not associated with any pulmonary function variables. Finally, the positive correlation between bone mass and FVC (ρ = 0.551, *p* < 0.001; “moderate to good”) and FEV1 (ρ = 0.571, *p* < 0.001; “moderate to good”) highlighted the importance of bone density for pulmonary capacity.

## 4. Discussion

This study aimed to examine the relationships between body composition, bone mineral density (BMD), muscle strength, and pulmonary function in firefighters from the Porto district. The findings reveal significant associations among these variables, offering important insights into the physical profile of these professionals and highlighting potential implications for their physical fitness and health.

The body composition findings indicate that the average BMI of both male and female participants falls into the overweight range, a trend commonly observed in firefighters due to the high physical demands of their profession [20]. However, BMI should be interpreted with caution, as it does not distinguish between lean mass and body fat [21]. Firefighters often possess increased muscle mass, which may partially explain the elevated BMI values. The prevalence of obesity observed in 36.4% of men and 12.5% of women is concerning, given the established link between excess body fat and various health risks [22]. In particular, excess weight can negatively impact physical fitness, especially in tasks requiring strong cardiovascular fitness and muscular endurance, and increases the risk of musculoskeletal injuries during activities such as victim rescues and handling heavy equipment [23].

To mitigate these risks, interventions focused on reducing body fat percentage while preserving lean mass are essential. Exercise and nutritional programs that emphasize weight loss while maintaining muscle mass can enhance both physical fitness and long-term health outcomes. A combination of aerobic and resistance training is particularly effective, as it promotes fat loss and muscle strengthening, ultimately improving physical fitness and reducing health risks [24,25,26].

The analysis of bone mineral density yielded noteworthy results. While the BMD of both male and female participants was within expected age-related ranges, women showed slightly higher bone density. This contrasts with the typical pattern of BMD decline after middle age [27]. Firefighting tasks, which involve heavy lifting and repetitive movements, may contribute to the preservation of bone density, particularly in men, who tend to maintain higher BMD until their third decade of life [28,29]. However, the cumulative physical stress and the potential for inadequate nutrition during rest periods could accelerate BMD loss, potentially affecting firefighters’ long-term bone health [30]. Thus, interventions aimed at preserving bone health are critical. Programs focusing on calcium and vitamin D supplementation, along with regular physical activity, may help prevent bone loss in this population [29,31].

Handgrip strength, a key indicator of overall muscular strength, showed a positive correlation with lean body mass (ρ = 0.55; *p* < 0.001), as expected, since muscle strength is closely tied to lean tissue mass [32]. This finding underscores the importance of maintaining sufficient lean mass to ensure optimal physical fitness. Muscular strength is essential for firefighters, whose duties require explosive strength and muscular endurance, particularly during emergency operations such as firefighting and rescue efforts [33]. Notably, over 20% of participants demonstrated handgrip strength values below the risk threshold, indicating vulnerability to reduced physical fitness and an increased risk of injury. As muscular strength is directly associated with mobility and functionality, strength training interventions tailored to firefighters’ needs could significantly improve their fitness levels [34,35,36].

This study also examined pulmonary function, a critical factor for firefighter physical fitness [37]. Positive correlations between handgrip strength and spirometric parameters, specifically FEV1 and PEF (ρ = 0.528; *p* < 0.001 for FEV1; ρ = 0.457; *p* < 0.001 for PEF), highlight the relationship between muscular strength and respiratory function [38]. Firefighters are frequently exposed to pulmonary irritants, such as smoke and other pollutants, which can compromise lung function over time [39,40]. Chronic exposure to these toxins is known to reduce pulmonary capacity and increase the risk of developing chronic obstructive pulmonary disease (COPD) [40]. Maintaining muscular strength and lean body mass not only enhances overall physical fitness but may also protect against declines in cardiovascular and pulmonary health [41,42]. While the majority of participants demonstrated normal pulmonary function, 5.8% showed signs of obstructive patterns, necessitating closer monitoring. Early interventions, including smoking cessation programs and proper use of respiratory protective equipment, are critical in reducing the risk of developing chronic pulmonary conditions such as COPD, which is highly prevalent among workers exposed to environmental pollutants [43].

Overall, the results of this study underscore the importance of interventions targeting body composition, muscular strength, bone health, and pulmonary function for the well-being of firefighters. Maintaining an optimal body composition focusing on reducing excess body fat and preserving lean mass can improve both physical fitness and long-term health outcomes. Regular strength training and weight management programs, along with ongoing pulmonary function monitoring, should be incorporated into firefighter wellness initiatives [24]. The positive correlation between muscular strength and pulmonary function suggests that resistance training not only benefits muscular fitness but may also have a protective effect on respiratory health. Additionally, health promotion strategies addressing smoking risks and the proper use of personal protective equipment are essential in safeguarding firefighter health.

The primary strengths of this study lie in the use of robust methodologies and precise, accurate equipment with good reproducibility. These include DEXA for body composition analysis, the handgrip dynamometer for evaluating muscular strength, and the portable spirometer for assessing pulmonary function. These tools facilitated a detailed and reliable evaluation of the variables under investigation, providing valuable insights into the health and physical fitness of firefighters. However, certain limitations must be acknowledged. As this is an ongoing project, challenges in obtaining detailed individual lifestyle data (e.g., nutritional profile and physical activity levels outside of work) and the incidence of various work-related incidents have precluded a more comprehensive analysis of the fitness profile of firefighters in northern Portugal. Furthermore, a comparison between firefighters and the general population of Porto would be informative; however, data for the overall population is not available for this analysis, which limits our ability to draw broader conclusions. The ability to stratify firefighters according to their specific roles could further refine the understanding of occupational demands and their impacts on health. Additionally, while handgrip strength was used as a measure of muscle strength, it primarily assesses grip and forearm strength, which may not fully reflect the multi-joint and functional strength required for firefighting tasks. Isokinetic muscle testing using the Biodex system (Biodex System 4 Pro, Biodex Medical Systems, Shirley, NY, USA), which allows for the assessment of strength and endurance across multiple muscle groups in both upper and lower body movements, could provide a more comprehensive evaluation of the physical demands placed on firefighters.

## 5. Conclusions

This study highlights the critical role of body composition in firefighter health and physical fitness. Maintaining an appropriate body composition, with a focus on lean mass preservation and fat reduction, is closely linked to muscle strength and pulmonary function. Excess body fat negatively impacts muscle strength, potentially reducing fitness levels necessary for the physically demanding tasks of firefighting. Conversely, handgrip strength was positively associated with better pulmonary function, emphasizing the importance of physical conditioning for respiratory health. Interventions targeting body composition, such as weight management and strength training, are essential for optimal firefighter physical fitness and long-term health. Strength training, in particular, may slow the decline in physical and respiratory function over time, improving both safety and effectiveness in high-demand environments. While robust assessment tools, such as DEXA, handgrip dynamometers, and spirometers, provided reliable insights, future studies should consider lifestyle factors, including physical activity outside of work and tobacco use, as well as stratifying firefighters by specific roles to better understand how occupational demands affect health. Additionally, programs focusing on strength training and pulmonary rehabilitation may further enhance physical fitness and quality of life for firefighters.

## Figures and Tables

**Table 1:** Characteristics of the study sample: demographic, anthropometric, bone health, and pulmonary function measures.

Variables	Sex	n	Mean ± SD	Median	Percentile Ranges			**SW (*p*-Value)**
					25%	50%	**75%**	
**Anthropometric and body composition**								
Age (years)	Female	8	31.4 ± 5.9	31.0	26.5	31.0	36.3	0.937 (0.58)
	Male	55	34.2 ± 7.4	35.0	29.0	35.0	39.5	0.979 (0.458)
Height (cm)	Female	8	163.0 ± 5.7	161.5	160.0	161.5	167.0	0.938 (0.587)
	Male	55	173.9 ± 7.6	173.0	168.5	173.0	178.5	0.961 (0.074)
Body weight (kg)	Female	8	66.1 ± 7.4	66.5	60.5	66.5	70.0	0.979 (0.958)
	Male	55	83.7 ± 14.1	85.0	71.5	85.0	94.5	0.959 (0.056)
BMI (kg/m^2^)	Female	8	25.0 ± 3.3	24.9	22.8	24.9	26.4	0.969 (0.887)
	Male	55	27.7 ± 4.7	26.9	24.2	26.9	31.9	0.949 (0.022)
BMI—normal weight	Female	4 (6.3%)	22.5 ± 1.9	22.4	21.3	22.4	23.6	0.982 (0.914)
	Male	15 (23.8%)	21.9 ± 1.7	21.9	20.8	22.5	23.1	0.950 (0.530)
BMI—overweight	Female	3 (4.8%)	26.4 ± 1.8	25.7	25.4	25.7	27.1	0.890 (0.355)
	Male	20 (31.7%)	26.9 ± 1.6	26.4	25.7	26.4	28.0	0.877 (0.016)
BMI—obesity	Female	1 (1.6%)	30.5	30.5	30.5	30.5	30.5	-
	Male	20 (31.7%)	32.9 ± 1.4	33.5	31.7	33.5	34.0	0.900 (0.041)
Total lean mass (kg)	Female	8	42.5 ± 3.7	42.6	40.9	42.6	44.9	0.941 (0.619)
	Male	55	61.5 ± 8.3	61.5	54.6	61.5	67.0	0.962 (0.083)
Total bone mineral content (kg)	Female	8	2.2 ± 0.3	2.1	2.0	2.1	2.3	0.873 (0.16)
	Male	55	2.6 ± 0.4	2.6	2.4	2.6	2.8	0.961 (0.069)
Total fat-free mass (kg)	Female	8	44.7 ± 3.9	44.8	42.8	44.8	47.5	0.947 (0.685)
	Male	55	64.1 ± 8.5	64.1	57.1	64.1	69.8	0.962 (0.079)
Total fat mass (kg)	Female	8	21.0 ± 5.0	20.1	17.6	20.1	22.6	0.928 (0.499)
	Male	55	19.0 ± 7.5	19.6	12.1	19.6	24.9	0.954 (0.036)
Body fat percentage (%)	Female	8	31.7 ± 4.5	31.5	29.6	31.5	33.9	0.952 (0.729)
	Male	55	22.1 ± 6.0	23.3	17.0	23.3	27.4	0.956 (0.044)
**Bone Health**								
Femoral neck area (cm^2^)	Female	3	4.7 ± 0.5	4.9	4.5	4.9	5.0	0.846 (0.23)
	Male	24	5.5 ± 0.3	5.5	5.2	5.5	5.6	0.929 (0.093)
Femoral neck bone mass (g)	Female	3	4.0 ± 0.3	4.1	3.9	4.1	4.1	0.879 (0.321)
	Male	24	5.0 ± 0.7	4.8	4.5	4.8	5.3	0.916 (0.047)
Femoral neck BMD (g/cm^2^)	Female	3	0.8 ± 0.0	0.8	0.8	0.8	0.9	0.75 (<0.001)
	Male	24	0.9 ± 0.1	0.9	0.8	0.9	1.0	0.84 (0.001)
Femoral neck T-score	Female	2	0.1 ± 0.4	0.1	−0.1	0.1	0.2	-
	Male	21	−0.3 ± 0.9	−0.5	−0.7	−0.5	−0.2	0.766 (<0.001)
Femoral neck Z-score	Female	3	0.1 ± 0.3	0.1	−0.1	0.1	0.2	0.987 (0.78)
	Male	24	0.1 ± 0.9	−0.2	−0.5	−0.2	0.4	0.845 (0.002)
**Muscle strength**								
HG right hand (kg)	Female	8	31.2 ± 3.5	31.6	29.1	31.6	32.9	0.994 (0.999)
	Male	55	47.3 ± 8.4	47.4	42.4	47.4	51.0	0.964 (0.098)
HG left hand (kg)	Female	8	30.4 ± 4.4	30.7	29.0	30.7	32.0	0.961 (0.815)
	Male	55	47.0 ± 7.6	46.2	42.6	46.2	52.1	0.934 (0.005)
HG peak (kg)	Female	8	31.6 ± 3.6	31.6	29.9	31.6	34.0	0.99 (0.996)
	Male	55	48.7 ± 7.8	48.5	44.4	48.5	52.7	0.965 (0.112)
**Pulmonary function**								
FVC (L)	Female	8	4.1 ± 0.4	4.1	3.8	4.1	4.4	0.948 (0.693)
	Male	55	5.3 ± 1.2	5.2	4.6	5.2	5.9	0.872 (<0.001)
FVC (%)	Female	8	116.1 ± 10.5	118.6	111.6	118.6	122.5	0.892 (0.243)
	Male	55	111.4 ± 18.6	109.7	98.5	109.7	120.1	0.89 (<0.001)
FEV1 (L)	Female	8	3.4 ± 0.3	3.4	3.1	3.4	3.5	0.897 (0.269)
	Male	55	4.3 ± 0.8	4.2	3.9	4.2	4.6	0.972 (0.233)
FEV1 (%)	Female	8	110.6 ± 9.0	111.4	104.8	111.4	116.6	0.973 (0.919)
	Male	55	106.9 ± 14.9	107.7	95.5	107.7	113.4	0.977 (0.382)
TI	Female	8	83.0 ± 5.6	83.9	79.3	83.9	86.0	0.978 (0.95)
	Male	55	80.6 ± 6.3	81.1	76.0	81.1	85.0	0.982 (0.582)
TI (%)	Female	8	95.8 ± 6.3	97.6	92.3	97.6	98.7	0.942 (0.627)
	Male	55	97.3 ± 7.4	98.7	92.8	98.7	102.5	0.98 (0.48)
PEF (L/s)	Female	8	6.1 ± 1.0	6.2	5.1	6.2	6.9	0.921 (0.442)
	Male	55	9.2 ± 2.0	9.1	8.1	9.1	10.6	0.985 (0.737)
PEF (%)	Female	8	88.8 ± 17.4	91.2	73.4	91.2	100.0	0.942 (0.635)
	Male	55	98.1 ± 21.7	99.2	81.2	99.2	112.3	0.988 (0.851)
**Smoking status**								
Yes	Female	3 (4.8%)	-	-	-	-	-	-
	Male	27 (42.9%)	-	-	-	-	-	-
No	Female	5 (7.9%)	-	-	-	-	-	-
	Male	20 (31.7%)	-	-	-	-	-	-
Ex-smokers	Female	0	-	-	-	-	-	-
	Male	8 (12.7%)	-	-	-	-	-	-

Notes: Data are presented as mean and standard deviation (SD), median, and percentile ranges. The normality was checked through the Shapiro–Wilk (SW) test. Abbreviations: body mass index (BMI); bone mineral density (BMD); handgrip (HG); forced vital capacity absolute (FVC); forced vital capacity percentage (FVC%); forced expiratory volume in 1s absolute (FEV1); forced expiratory volume in 1s percentage (FEV1%); Tiffeneau index (TI); Tiffeneau index percentage (TI%); peak expiratory flow absolute (PEF); peak expiratory flow percentage (PEF%). Statistical significance was considered when *p* < 0.05.

**Table 2:** Partial correlations between the variables of body composition, muscle strength, and lung function.

Partial Correlation		Body Weight (kg)	HG Right Hand (kg)	HG Left Hand (kg)	HG Peak (kg)	Total Bone Mineral Content (kg)	Total Fat Mass (kg)	Total Lean Mass (kg)	Total Fat-Free Mass (kg)	Total Fat (%)
Body weight (kg)	Spearman’s rho	—								
	*p*-value	—								
HG right hand (kg)	Spearman’s rho	0.364	—							
	*p*-value	0.005	—							
HG left hand (kg)	Spearman’s rho	0.371	0.796	—						
	*p*-value	0.004	<0.001	—						
HGPeak (kg)	Spearman’s rho	0.4	0.917	0.928	—					
	*p*-value	0.002	<0.001	<0.001	—					
Total bone mineral content (kg)	Spearman’s rho	0.662	0.286	0.379	0.343	—				
	*p*-value	<0.001	0.028	0.003	0.008	—				
Total fat mass (kg)	Spearman’s rho	0.395	−0.109	−0.184	−0.147	0.12	—			
	*p*-value	0.002	0.413	0.164	0.266	0.367	—			
Total lean mass (kg)	Spearman’s rho	0.865	0.478	0.535	0.551	0.645	−0.02	—		
	*p*-value	<0.001	<0.001	<0.001	<0.001	<0.001	0.883	—		
Total fat-free mass (kg)	Spearman’s rho	0.866	0.487	0.547	0.564	0.662	−0.021	0.998	—	
	*p*-value	<0.001	<0.001	<0.001	<0.001	<0.001	0.877	<0.001	—	
Body fat percentage (%)	Spearman’s rho	0.022	−0.285	−0.346	−0.325	−0.149	0.855	−0.352	−0.352	—
	*p*-value	0.869	0.029	0.007	0.012	0.262	<0.001	0.006	0.006	—
FVC (L)	Spearman’s rho	0.559	0.419	0.483	0.465	0.311	0.285	0.471	0.48	0.007
	*p*-value	<0.001	<0.001	<0.001	<0.001	0.017	0.029	<0.001	<0.001	0.959
FVC (%)	Spearman’s rho	0.175	0.283	0.387	0.332	0.028	0.098	0.12	0.125	−0.036
	*p*-value	0.184	0.03	0.002	0.01	0.832	0.459	0.364	0.345	0.789
FEV1 (L)	Spearman’s rho	0.507	0.476	0.529	0.529	0.322	0.132	0.506	0.512	−0.147
	*p*-value	<0.001	<0.001	<0.001	<0.001	0.013	0.318	<0.001	<0.001	0.268
FEV1 (%)	Spearman’s rho	0.147	0.33	0.415	0.384	0.059	−0.031	0.186	0.19	−0.167
	*p*-value	0.268	0.011	0.001	0.003	0.658	0.815	0.159	0.15	0.206
TI	Spearman’s rho	−0.148	0.055	−0.001	0.061	0.036	−0.285	0.032	0.035	−0.24
	*p*-value	0.264	0.679	0.994	0.645	0.785	0.029	0.808	0.791	0.068
TI (%)	Spearman’s rho	−0.072	0.081	0.044	0.101	0.083	−0.239	0.11	0.112	−0.222
	*p*-value	0.587	0.543	0.741	0.448	0.531	0.068	0.408	0.397	0.09
PEF (L/s)	Spearman’s rho	0.197	0.396	0.371	0.457	0.157	−0.001	0.209	0.218	−0.158
	*p*-value	0.135	0.002	0.004	<0.001	0.236	0.992	0.111	0.097	0.232
PEF (%)	Spearman’s rho	−0.012	0.309	0.284	0.36	0.012	−0.06	0.003	0.011	−0.149
	*p*-value	0.93	0.017	0.029	0.005	0.929	0.651	0.979	0.933	0.26
Femoral neck area (cm^2^)	Spearman’s rho	0.513	0.306	0.435	0.387	0.677	0.142	0.609	0.585	−0.028
	*p*-value	0.012	0.155	0.038	0.068	<0.001	0.517	0.002	0.003	0.899
Femoral neck bone mineral content (g)	Spearman’s rho	0.338	0.392	0.369	0.41	0.645	0.066	0.436	0.431	0.019
	*p*-value	0.115	0.064	0.083	0.052	<0.001	0.764	0.038	0.04	0.933
Femoral neck BMD (g/cm^2^)	Spearman’s rho	−0.029	0.221	0.13	0.196	0.197	−0.016	0.046	0.053	0.083
	*p*-value	0.895	0.312	0.556	0.37	0.369	0.944	0.834	0.81	0.708
Femoral neck T-score	Spearman’s rho	0.099	0.209	0.139	0.194	0.246	0.057	0.135	0.147	0.15
	*p*-value	0.686	0.391	0.571	0.427	0.31	0.816	0.583	0.55	0.541
Femoral neck Z-score	Spearman’s rho	0.039	0.295	0.2	0.265	0.321	−0.081	0.162	0.16	0.019
	*p*-value	0.859	0.172	0.36	0.222	0.135	0.713	0.46	0.466	0.931

Notes: Controlled by “age”, “sex”, “BMI”, and “smoking status”. Abbreviations: body mass index (BMI); bone mineral density (BMD); handgrip (HG); forced vital capacity absolute (FVC); forced vital capacity percentage (FVC%); forced expiratory volume in 1s absolute (FEV1); forced expiratory volume in 1s percentage (FEV1%); Tiffeneau index (TI); Tiffeneau index percentage (TI%); peak expiratory flow absolute (PEF); peak expiratory flow percentage (PEF%).

**Table 3:** Partial correlations between the variables of trunk body composition and lung function.

Partial Correlation		FVC	FVC%	FEV1	FEV1%	TI	TI%	FEP	FEP%
Trunk total mass (kg)	Spearman’s rho	0.610	0.264	0.540	0.213	−0.165	−0.086	0.202	0.011
	*p*-value	<0.001	0.044	<0.001	0.105	0.212	0.517	0.124	0.932
Trunk fat mass (kg)	Spearman’s rho	0.280	0.103	0.118	−0.03	−0.297	−0.255	−0.044	−0.086
	*p*-value	0.032	0.437	0.372	0.824	0.022	0.051	0.742	0.518
Trunk lean mass (kg)	Spearman’s rho	0.555	0.241	0.574	0.284	0	0.076	0.254	0.06
	*p*-value	<0.001	0.066	<0.001	0.029	0.999	0.565	0.053	0.65
Trunk bone mineral content (kg)	Spearman’s rho	0.551	0.231	0.571	0.275	0.003	0.08	0.254	0.059
	*p*-value	<0.001	0.079	<0.001	0.035	0.983	0.548	0.052	0.659

Notes: Controlled by “age”, “sex”, “BMI”, and “smoking status”. Abbreviations: body mass index (BMI); forced vital capacity percentage (FVC%); forced expiratory volume in 1s absolute (FEV1); forced expiratory volume in 1s percentage (FEV1%); Tiffeneau index (TI); Tiffeneau index percentage (TI%); peak expiratory flow absolute (PEF); peak expiratory flow percentage (PEF%).

## Data Availability

The data presented in this study are available upon request from the corresponding author. The data are not publicly available due to the inclusion of information that could compromise the privacy of study participants.
